# Perspectives of Urban Corner Store Owners and Managers on Community Health Problems and Solutions

**DOI:** 10.5888/pcd13.160172

**Published:** 2016-10-13

**Authors:** Victoria L. Mayer, Candace R. Young, Carolyn C. Cannuscio, Allison Karpyn, Sarah Kounaves, Emily Strupp, Kevin McDonough, Judy A. Shea

**Affiliations:** Author Affiliations: Candace R. Young, The Food Trust, Philadelphia, Pennsylvania; Carolyn C. Cannuscio, Department of Family Medicine and Community Health, Perelman School of Medicine, and Center for Public Health Initiatives, University of Pennsylvania, Philadelphia, Pennsylvania; Allison Karpyn, The Center for Research in Education and Social Policy, University of Delaware, Newark, Delaware; Sarah Kounaves, Emily Strupp, Kevin McDonough, Department of Family Medicine and Community Health, Perelman School of Medicine, University of Pennsylvania, Philadelphia, Pennsylvania; Judy A. Shea, Division of General Internal Medicine, Department of Medicine, Perelman School of Medicine, University of Pennsylvania, Philadelphia, Pennsylvania.

## Abstract

**Introduction:**

Urban corner store interventions have been implemented to improve access to and promote purchase of healthy foods. However, the perspectives of store owners and managers, who deliver and shape these interventions in collaboration with nonprofit, government, and academic partners, have been largely overlooked. We sought to explore the views of store owners and managers on the role of their stores in the community and their beliefs about health problems and solutions in the community.

**Methods:**

During 2013 and 2014, we conducted semistructured, in-depth interviews in Philadelphia, Pennsylvania, and Camden, New Jersey, with 23 corner store owners/managers who participated in the Healthy Corner Store Initiative spearheaded by The Food Trust, a nonprofit organization focused on food access in low-income communities. We oversampled high-performing store owners.

**Results:**

Store owners/managers reported that their stores served multiple roles, including providing a convenient source of goods, acting as a community hub, supporting community members, working with neighborhood schools, and improving health. Owners/managers described many challenging aspects of running a small store, including obtaining high-quality produce at a good price and in small quantities. Store owners/managers believed that obesity, diabetes, high cholesterol, and poor diet are major problems in their communities. Some owners/managers engaged with customers to discuss healthy behaviors.

**Conclusion:**

Our findings suggest that store owners and managers are crucial partners for healthy eating interventions. Corner store owners/managers interact with community members daily, are aware of community health issues, and are community providers of access to food. Corner store initiatives can be used to implement innovative programs to further develop the untapped potential of store owners/managers.

## Introduction

Chronic, diet-related diseases and obesity disproportionately affect people of low socioeconomic status and racial/ethnic minorities in the United States (1,2). Urban food environments play an important role in determining population-level obesity risk (3–5). Low-income neighborhoods are often characterized by having few supermarkets, many small stores with a poor selection of fruits and vegetables, and many fast-food outlets (3–7). Nonprofit organizations, community organizations, and state and local governments have developed interventions to improve access to healthy foods by developing new food outlets and modifying existing stores (8–10).

Small food stores (alternatively known as *corner stores*, *convenience stores*, or *bodegas*) have been the target of food environment interventions in several US cities, with some evidence of increased availability and sales of healthy foods and improvement in consumers’ knowledge about and behaviors surrounding healthy eating (10–12). The Food Trust, a nonprofit organization established in 1992, developed and implemented the Healthy Corner Store Initiative (HCSI), which provided education (eg, training on produce sourcing and storage), incentives to modify inventory, and equipment (eg, refrigerated coolers, storage racks for fresh produce) to owners/managers of small food stores. The HCSI launched in Philadelphia, Pennsylvania, in 2004 in cooperation with the Philadelphia Department of Public Health’s Get Healthy Philly initiative, and in Camden, New Jersey, in 2011, as part of the Healthy Camden program. Both cities have high proportions of racial/ethnic minority residents (64% Philadelphia, 95% Camden) and poor residents (25% Philadelphia, 38% Camden) and high rates of adult obesity (32% Philadelphia, 30% Camden) and diabetes (13% Philadelphia, 10% Camden) (13–16).

Although the success of these interventions depends on the influence and involvement of store owners/managers, little is known about their perspectives (10,17–20). We conducted in-depth interviews to 1) further understand how owners/managers view their communities’ health concerns and their stores’ community roles and 2) to understand the challenges of running corner stores.

## Methods

We developed an interview guide in English and Spanish and adapted it with input from The Food Trust’s field staff. After conducting 2 pilot interviews with store owners to identify potential challenges in flow and comprehension, the guide was changed modestly by adding clarifying probes. We conducted 23 semistructured interviews with active HCSI corner store owners/managers in Philadelphia and Camden. The interview guide addressed community concerns and health beliefs surrounding chronic diseases and diet, perceived role of the corner store in the community, and motivations for HCSI participation (Table 1). We also investigated the perceived impact, successes, and challenges of the HCSI in a separate analysis.

**Table 1:** Interview Guide: Areas of Inquiry and Sample Questions for Corner Store Owner and Manager Interviews, Philadelphia, Pennsylvania, and Camden, New Jersey, 2013–2014

Area of Inquiry	Sample Questions/Probes
Store operations^a^	Tell me about when you started running this store.
Role of store in community^a^	As a store owner, what is your role in the community? What do you see as the role of your store in the community?
Healthy Corner Store Initiative participation and motivations^a^	What do you think about the healthy corner store program? What made you join the program?
Program effects on store	What changes have you noticed in your store since joining the program? (both in what you stock and among customers) Have you seen any changes in the number of customers you have? How have the products that you sell changed, if at all? Has the type of equipment in your store changed?
Program successes, challenges, and recommendations	What have the program successes been? What about the program has worked well in the store? What challenges or difficulties have you experienced with the program? What doesn’t work well? What advice do you have for us to improve the program?
Community health^a^	What are some major health problems in this community? Does the store have a role in the health of the community?

a These areas are the focus of the article.

At the time of the study (September 2013 through January 2014), The Food Trust was working with 14 stores in Camden, New Jersey, and more than 600 stores in Philadelphia, Pennsylvania. We recruited a purposeful sample of stores from a list that included the 14 Camden stores and 25 top performing Philadelphia stores. “Top performing” was defined as having completed all available training sessions and demonstrated expanded inventory of recommended healthier items, along with full implementation of healthy food marketing efforts provided by the program. We selected top performing stores to explore potential attitudes and behaviors of high-achieving HCSI participants — an approach informed by the positive deviance model, which focuses on uncommon and successful strategies of individuals in a group that consists of people facing similar challenges (21). Stores were selected from the combined list to generate a geographically diverse sample. The team iteratively reviewed interview findings during data collection and reached thematic saturation after completing 23 interviews.

Investigators (V.M. and C.Y.) together approached store owners/managers in their stores, recruited them, obtained written informed consent, interviewed participants in English or Spanish (C.Y. only), and recorded notes. The investigators (V.M. and C.Y.) alternated between conducting interviews and listening, and between observing and taking notes. In many cases, the 30- to 60-minute semistructured interview occurred that day; in a few cases, interviews were scheduled for a future date. Participants received a $25 Visa gift card. Interviews in Spanish and English were audio-recorded and transcribed in English by a HIPAA (Health Information Portability and Accountability Act)–compliant company.

Qualitative coding and data interpretation followed an iterative process guided by grounded theory. The 2 interviewers independently developed initial coding schemes on the basis of close reading of the data. These coding schemes were then compared and reconciled into a single version that was further discussed and revised with the full team. Three research team members (K.M., S.K., and E.S.) tested the coding scheme by coding 1 interview together and 5 separately. Minor adjustments were made, and a final coding scheme was reached. Research staff then coded the remaining 17 interviews (1 researcher per interview). The team collaborated to summarize data coded to each category and to identify cross-cutting themes (22,23). We used NVivo version 10 (QSR International) to analyze data. The study was approved by the institutional review board at the University of Pennsylvania.

## Results

### Demographics

We interviewed owners/managers at 23 stores: 6 in Camden out of 8 approached (75%) and 17 in Philadelphia out of 21 approached (81%) (Table 2). Of all participants, 65% were male. Most (61%) were aged 26 to 40 years. Most (83%) participants identified as Hispanic or Latino, and many (48%) were originally from the Dominican Republic. The range of years working in the current corner store was broad (4 months to 39 years).

**Table 2:** Demographics of Corner Store Owners/Managers (N = 23) in Philadelphia, Pennsylvania, and Camden, New Jersey, 2013–2014

Characteristic	Value^a^
**City of store: Philadelphia, PA**	17 (74)
**City of store: Camden, NJ**	6 (26)
**Female**	8 (35)
**Age, y**	
18–25	1 (4)
26–40	14 (61)
41–65	7 (30)
>65	0
Missing	1 (4)
**Race/ethnicity**	
Hispanic or Latino	19 (83)
Asian	1 (4)
Arab American	1 (4)
White	2 (9)
**Country of origin**	
Dominican Republic	11 (48)
United States	4 (17)
Mexico	2 (9)
Korea	1 (4)
Puerto Rico	2 (9)
Nicaragua	1 (4)
Turkey	1 (4)
Yemen	1 (4)
**Mean no. of years at current store (range)**	6 (4 months–39 years)
**Mean no. of years working at any corner store (range)**	11 (1–39 years)

a Values expressed as no. (%) unless otherwise indicated.

### Stores as community support

We identified several cross-cutting themes (Box). Overall, the owners/managers agreed the role of the corner store was to provide for the local community. Most store owners/managers explained their main priority was to provide whatever goods their surrounding community needed and wanted; as one owner stated, “Well, it’s a convenient store; it’s a convenience for the neighborhood, that they have everything close and accessible.” They emphasized the importance of variety and of providing goods that were otherwise difficult to find in the neighborhood because of a lack of nearby supermarkets.

**Box. Cross-Cutting Themes: Perceptions of Store Owners and Managers in Philadelphia, Pennsylvania, and Camden, New Jersey, 2013–2014**

**Table:** 

Cross-Cutting Theme
**Corner stores serve as community support.**
Owners/managers struggled with small profit margins; they rely on family and food assistance programs (SNAP/WIC) to support their businesses.
Owners/managers faced challenges in procuring affordable, regular distribution of produce.
Owners/managers identified obesity, diabetes, smoking, high cholesterol, and poor eating habits as important health problems in the community.
Some owners/managers incorporated activities to address perceived health problems into their businesses, including modifying product inventory and verbally encouraging customers to make different choices.

Abbreviations: SNAP, Supplemental Nutrition Assistance Program; WIC, Special Supplemental Nutrition Program for Women, Infants, and Children.

However, interviewees also mentioned several additional roles, exemplified by one owner’s statement that “. . . as owners, what we want is to sell, but at the same time, we would like to help the community.” Several owners/managers reported their stores serve as a gathering place, a social center, and communication hub, for the surrounding community (3 of 23 stores). One owner said that he “serves as a communicator,” because he has an area where people can post advertisements or messages. Another likened his store to “a bar for old ladies,” explaining, “. . . they’ll just come in and hang out and gossip . . . it’s a social thing.”

Many store owners/managers (6 of 23) discussed making charitable donations to community organizations or customers: “Sometimes you treat people like family, I guess. Time to hear their problems or situations, or sometimes people come in, they don’t have no food and, you know, you give them food, I guess.” Another owner described charitable giving as an example of how the store fits into the community: “Whenever there is something the whole community does, we always be the first to always participate in donations and stuff like that to keep it in the community, basically — financially, and emotionally, and everything.”

Many owners/managers also reported a role for their store in improving the health and overall well-being of the people, particularly children, in their communities (8 of 23). An owner explained, “We have tried to get the community to eat healthier and especially the kids. We want everybody to eat healthier, but the kids who are growing up, they are the ones who need to have those healthy eating habits.” These owners/managers perceived that selling certain products, especially fruits and vegetables, helped the health of their customers. This desire to promote healthy eating was often at odds with other available offerings:

The store . . . sells some junk food that the community wants. We cannot stop selling it, because that’s what the customers want, but we also give them another option to show them that, “Let’s reduce this, let’s do this more,” and some people like it, some people ignore it.

Several owners/managers described store activities aimed at children in the community. Four stores collaborated with nearby schools by supplying food for events, denying service to school-aged children after 7:40 am to ensure they were not late for school, and providing a safe haven and a telephone in case of emergency. One owner described the important role she and her husband feel they have played for young people in the community: “A lot of young people in this area are suffering . . . more than anything, our goal is to encourage them to do the right thing.”

### Small profit margins mean reliance on family, SNAP/WIC

A second prominent theme identified in our interviews was the challenge of maintaining a small business with small profit margins. Owners relied heavily on family. One owner explained, “All the family works here.” Most owners/managers worked every day of the week, sometimes 16 to 17 hours per day.

Store owners/managers noted the effect on their businesses of food assistance benefits (SNAP, the Supplemental Nutrition Assistance Program, and WIC, the Special Supplemental Nutrition Program for Women, Infants, and Children). One store owner said that if the government got rid of SNAP, they would have to close down. Another owner reported that fruit sales were directly related to when people received benefits: “Between the 2nd and the 20th of the month, we sell a lot of fruit because we have the coupons, the WIC checks.”

### Challenges of affordable, regular distribution of produce

Almost all store owners/managers discussed the challenges of purchasing fresh fruits and vegetables at prices that make carrying these items profitable. Purchasing at a wholesale market is one of the best and cheapest ways to get produce, but quantities are big: “They’re too big, so what happens is you buy them, if you don’t sell it, the other part goes bad.” To avoid spoilage, owners/managers sometimes purchased smaller quantities at a supermarket, but prices are higher. Another solution for produce sourcing that many used was an independent delivery service. The delivery person calls the store to take their produce order and delivers produce from a wholesale market directly to the store, charging a mark-up on the produce.

### Views on community health

Owners/managers consistently observed that obesity, especially among children, diabetes, and high cholesterol were problems in the community. Poor eating habits were often described as a major health problem: “[People in the neighborhood] go to the corner stores, and they buy just a bunch of cakes and chips and you know, just junk food in general.” Many seemed personally frustrated by the unhealthy choices of their customers:

There are people who come here that ask for [coffee], with 7 sugar packets — it’s almost half the cup of sugar, and you put that in one coffee, that’s too much. . . . That’s diabetes you’re looking for, and there are a lot of diabetic people around here.

Participants saw poor decision-making on the part of community members, lack of education, economic conditions, and the food environment as causes of health problems. Although several owners/managers commented that individuals ate unhealthy foods because they “don’t care,” others pointed to a lack of education about nutrition. Others believed that choices were dictated by economics; one explained that unhealthy eating occurs

. . . because people in this neighborhood can only afford so much. They’re gonna choose a bag of potato chips and a cake that costs less than fruit. If I have to pay so much money for a fruit, I’m gonna leave that there, and I’m gonna go for the cake.

Store owners/managers addressed health behaviors with customers with whom they were familiar and with children (12 of 23). Some made minor suggestions; others would tell customers what they should or should not be doing regarding diet and smoking: “We deal with them every day, and we see they are sick. A man who is our customer and who has high sugar was eating pastry the other day so I told him not to eat that.”

Owners that addressed health with customers seemed most comfortable asserting the message to quit smoking:

Well any chance I get to talk, like to suggest that people quit, I do. I don’t sell them cheap, because I don’t wanna sell a lot of them and I don’t advertise them at all. I have to have them, because it’s just expected out of a corner store in this area.

Another owner developed rapport with a particular customer and delivered a targeted message: “She [was] always coming in here and buy a pack of Merits. I’m like, you cannot be smoking.”

### Shifts in marketing and product inventory to address health

A few store owners elected not to sell tobacco products because of known health risks, which they sometimes experienced first-hand (2 of 23): “We don’t sell cigarettes either. . . . I mean, they’re unhealthy, and we weren’t making money on them. . . . That’s how I got cancer, because I did smoke.” Two other owners reduced tobacco marketing or raised tobacco prices to discourage smoking (2 of 23).

These owners acknowledged that by cutting back or eliminating unhealthy items such as tobacco and candy they were risking losing customers and having lower sales. However, they accepted this as a calculated risk and found that customers adapted or went to another store. One owner described changes after she stopped carrying candy:

Now, like, they come in, grab a banana . . . they’ll come in and they’ll grab a piece of fruit instead of grabbing candy, or they get a pizza, pretzel, which is much healthier than candy. . . .You just get different clientele, because people that want candy go to a different store. If they want candy and cigarettes, we tell them go to the store down there.

Store owners/managers who were comfortable speaking to customers about health issues only did so with “regulars” and with children. No store owners/managers were willing to tell unknown adults what to purchase or eat:

It depends if I already know you. But if you’re new, I will not try to, because I’m in business to make money. So by me trying to change you, you might say, “Well I don’t want this, I’ll put it back and go somewhere else.” So I lost a customer.

Many store owners/managers also talked about the limitations of what they could do. One owner summarized this belief, saying, “If they want to live that kind of life, that’s fine. I can’t do anything about it.”

## Discussion

This study investigated the perspectives of store owners/managers who participated in the HCSI in low-income neighborhoods of Philadelphia and Camden, with a focus on high-performing Philadelphia stores that demonstrated health-relevant changes during the intervention period. Findings shed light on 1) how owners/managers viewed their communities’ health concerns and their stores’ community roles, and 2) the perceived social and economic challenges of running corner stores. In addition to providing a convenient source of a variety of goods for community residents, owners believed that the store could and should play a role in improving health and in supporting community members and neighborhood schools. Store owners/managers believed that smoking, diabetes, and poor diet are major problems in their community. However, owners/managers indicated challenges involved in procuring quality produce at a good price in small quantities. They also described the importance of SNAP and WIC as a source of income. To encourage health among their customers, some owners/managers expressed an interest in shifting product availability and engaging customers.

Previous research, motivated by an ecological framework, has evaluated and described small food store interventions as programs that act on physical environments and on the consumer nutrition environment to influence what people eat (10,11,17,19,24,25). Our findings indicate that the individual store owner is an essential component of this setting. Owners’ beliefs appear to have measurable influence on store practices, such as not selling cigarettes and encouraging children to purchase and eat fruit. These findings support lessons learned from several other US cities with active corner store interventions that carefully considering store owner/managers’ perspectives is essential to an intervention’s success (9).

Our findings also show that owners/managers believe their businesses are affected by produce distribution systems and food assistance programs. Anderson Steeves et al identified the lack of attention to the broader food production and distribution network as a key gap in food environment intervention research (8). Developing more affordable and feasible mechanisms for small food stores to source fresh produce could make it easier to stock more fruits and vegetables, a major aim of small food store interventions.

Our findings also show that corner store owners perceive themselves and their stores as community resources and may seek to positively influence customer health behaviors. Although previous work has shown the importance of the interaction between store owners/managers and customers (9,26), ours is the first study we know of to describe this relationship as potentially health promoting. This new and important finding suggests that some store owners/managers may act in an informal capacity to address health and could benefit from more formalized training, similar to the community health worker model and health promotion interventions in beauty salons and barbershops (27–30). These informal health worker models are effective for health promotion in multiple settings and could be applied to healthy corner store initiatives.

Our study has limitations. Our sample included store owners/managers who were most successful in implementing elements of the HCSI. Stores were purposefully sampled, so participants in this study probably have higher-than-average interest and motivation to address community health. More research of a representative sample can elucidate health-relevant beliefs and practices of the larger population of urban store owners/managers.

This study has implications for small food store interventions. Programs can recruit store owners/managers as community leaders and train them in health communications and outreach. Initiatives can also implement programming that further builds on corner stores as venues for health promotion. Corner store owners who are committed to community health promotion are essential to any such initiative.

This study documents the interest of corner store owners/managers in taking an active role in community health and the challenges of doing so while operating successful small businesses in economically challenged urban areas. Innovative strategies are needed to align these goals and further engage urban store owners as partners for health.
